# Supplementary material for: Detailed molecular and epigenetic characterization of the pig IPEC-J2 and chicken SL-29 cell lines
Source: iScience. 2023 Feb 20;26(3):106252. doi: 10.1016/j.isci.2023.106252 (PMC10018572; doi:10.1016/j.isci.2023.106252)
Supplement: Data S1. Complete homer output for identified motifs in Pig IPECJ-2, related to Table 2 — Homer motif analysis results for histone modifications H3K4me1, H3K4me3, H3K27ac, and enhancer elements of pig IPECJ2 cell line. P-values >1e-10 are possible false positives. Within each folder (e.g. peak_files_CTCF) are the html files showing the identified motifs when using homer (e.g. homerResults.html). [file mmc2.zip › S5/Pig_IPECJ_2/peak_files_H3K27ac/homerResults/motif1.similar.html]

motif1

## Information for motif1

T
G
C
A
G
A
C
T
A
C
G
T
A
C
G
T
A
G
C
T
G
T
A
C
T
A
G
C
G
A
T
C
A
T
G
C
A
T
C
G
  
Reverse Opposite:  

T
A
G
C
T
A
C
G
C
T
A
G
A
T
C
G
C
A
T
G
C
T
G
A
C
G
T
A
C
G
T
A
C
T
G
A
A
C
G
T
  

|  |  |
| --- | --- |
| p-value: | 1e-141 |
| log p-value: | -3.261e+02 |
| Information Content per bp: | 1.582 |
| Number of Target Sequences with motif | 26128.0 |
| Percentage of Target Sequences with motif | 49.69% |
| Number of Background Sequences with motif | 22907.5 |
| Percentage of Background Sequences with motif | 44.17% |
| Average Position of motif in Targets | 149.4 +/- 84.6bp |
| Average Position of motif in Background | 149.5 +/- 91.0bp |
| Strand Bias (log2 ratio + to - strand density) | 0.0 |
| Multiplicity (# of sites on avg that occur together) | 1.39 |
| Motif File: | file (matrix) reverse opposite |

### Similar de novo motifs found

|  |  |  |  |  |  |  |  |
| --- | --- | --- | --- | --- | --- | --- | --- |
| Rank | Match Score | Redundant Motif | P-value | log P-value | % of Targets | % of Background | Motif file |
| 1 | 0.701 | A T C G G A C T C A T G C G T A T C G A T G C A T G A C A G C T | 1e-111 | -256.814125 | 44.13% | 39.31% | motif file (matrix) |
| 2 | 0.769 | A T G C A T C G A T C G A T C G T C A G C G T A C G T A T G C A | 1e-65 | -150.231993 | 35.52% | 32.01% | motif file (matrix) |
| 3 | 0.688 | A C G T A C G T A G C T T C G A A T G C C G A T C T A G A C T G | 1e-64 | -148.906695 | 22.42% | 19.42% | motif file (matrix) |
| 4 | 0.602 | A T G C A T G C A T G C A C T G T C G A G C T A G T C A T G C A A T C G A T G C C A G T T G C A T A G C | 1e-53 | -124.224478 | 52.62% | 49.23% | motif file (matrix) |
| 5 | 0.614 | G A C T A C G T G C A T G C T A T G C A T C G A T A G C T A G C | 1e-49 | -113.901363 | 30.63% | 27.70% | motif file (matrix) |
